# Implementation Strategies for a Technology-Based Bullying Bystander Intervention (STAC-T): Mixed Methods Study

**DOI:** 10.2196/92450

**Published:** 2026-10-29

**Authors:** Diana M Doumas, Aida Midgett, Nathanial J Williams, Claudia Peralta

**Affiliations:** 1 Counselor Education Boise State University Boise, ID United States

**Keywords:** technology-based intervention, bullying bystander intervention, STAC-T, Stealing the Show, Turning it Over, Accompanying Others, Coaching Compassion, Technology Version, implementation strategies, “go-zone”

## Abstract

**Background:**

Nearly 1 in 5 students aged 12-18 years report being bullied in the United States. Both targets and students who witness bullying experience negative mental health outcomes. School-based programs are effective in decreasing negative outcomes associated with bullying; however, significant implementation barriers associated with in-person programs limit program adoption, fidelity, and sustainability. STAC-T (Stealing the Show; Turning it Over; Accompanying Others; Coaching Compassion; Technology Version), an interactive bullying bystander app, was developed to reduce implementation barriers. However, little is known about what will be required to successfully translate STAC-T into routine practice in schools. As a first step, we examined school personnel perspectives on school-specific implementation strategies that would be both highly important and feasible (“go-zone” strategies) for delivering STAC-T. Identifying specific implementation strategies can further support implementation and program outcomes. Although researchers have developed a taxonomy of 33 school-specific “go-zone” implementation strategies, there is no research examining these strategies specifically for STAC-T.

**Objective:**

The aim of this study was to identify implementation strategies for STAC-T using “go-zone” methodology. “Go-zone” methodology categorizes implementation strategies into four zones: (1) zone 1: high importance, high feasibility; (2) zone 2: low importance, high feasibility; (3) zone 3: low importance, low feasibility; and (4) zone: 4 high importance, low feasibility. Objectives included (1) identifying “go-zone” strategies for STAC-T and (2) identifying zone 4 strategies for STAC-T and understanding how to increase the feasibility of these strategies.

**Methods:**

A self-selected convenience sample of school counselors and school social workers (N=195) recruited from national professional organizations completed an online survey. A subsample (n=14) completed qualitative interviews. “Go-zone” methodology was used to identify zone 1 (“go-zone”) and zone 4 strategies. Thematic analysis was used to extract themes related to increasing the feasibility of zone 4 strategies.

**Results:**

Fifteen implementation strategies were classified as “go-zone” strategies. The three most important strategies were (1) *make implementation easier by removing burdensome documentation tasks*, (2) *develop a detailed implementation plan or blueprint*, and (3) *obtain and use student and family feedback*. The most feasible strategy was *distribute educational materials*. Four strategies were classified in zone 4: (1) *develop instruments to monitor and evaluate core components of the innovative or new practice*, (2) *conduct educational meetings*, (3) *build partnerships to support implementation*, and (4) *provide ongoing consultation*. Qualitative data revealed that feasibility of zone 4 strategies can be increased by keeping evaluative assessments short and simple, using various modes of communication for educational meetings, building partnerships with support from school administration, and enhancing consultation through hybrid or virtual meetings and appointing a leader.

**Conclusions:**

Findings represent a first step in identifying implementation strategies that can be used to inform STAC-T implementation. Directions for future research include testing the efficacy of these strategies on improving both implementation and program outcomes.

## Introduction

### Background

In the United States, 19.2% of students aged 12-18 years report being bullied at school [1] and 15.6% report being cyberbullied [2]. Bullying peaks in middle school with 26.3% of middle school students reporting school bullying victimization [1] and 36.7% reporting being cyberbullied [2]. Being a target of bullying carries significant mental health risks, including anxiety, posttraumatic stress [3], internalizing symptoms [4], depressive symptoms, nonsuicidal self-injury, suicidal ideation, and suicide attempts [3,5,6]. Internalizing symptoms [7-11], suicidal ideation [6,7,9], self-harm, and suicide attempts [6] are also associated with being a target of cyberbullying. Similarly, symptoms of anxiety and depression are reported by students who witness school bullying [12-14] and cyberbullying [15-17]. Thus, implementing effective-school based interventions to address bullying is imperative to reduce the negative associated mental health outcomes for both students who are targets of bullying and students who witness bullying as bystanders.

### School-Based Bullying Intervention Programs

Research indicates that comprehensive, school-wide bullying programs have small but significant effects on reducing bullying [18-20]; however, these programs are expensive, complex, time intensive, and require extensive training [21]. Many schools face implementation barriers related to logistical issues associated with training and providing counseling resources, limited funding, lack of a clear antibullying policy, a lack of priority set by local decision makers and principals of schools, and the presence of competing issues facing schools [22]. Additional barriers include training requirements, limited time, varying staff commitment, and school contextual factors [23]. Research indicates that the clinical outcomes of prevention and promotion program are 2-3 times better when programs are well implemented than when programs are poorly implemented [24]. Thus, implementation barriers may impact intervention outcomes and potentially explain the small effect sizes seen in bullying intervention research. Additionally, few comprehensive, school-based programs incorporate bystander training. Bystander training is important as up to 80% of students report witnessing bullying as bystanders [14]. Only 20%-30% of students who witness bullying, however, intervene [25,26]. One reason students do not intervene is that they do not know what to do when they witness a bullying situation [27]. Additionally, some bystanders who attempt to intervene in bullying engage in the bullying event in an aggressive, maladaptive manner [28]. Defending targets is also positively related to internalizing symptoms, including depression and anxiety [28,29]. Thus, bystander training (eg, teaching students who witness bullying to appropriately intervene) is a promising approach to bullying prevention to both reduce bullying and improve bystander mental health.

### The STAC Bullying Bystander Intervention

STAC (Stealing the Show; Turning it Over; Accompanying Others; Coaching Compassion) is a brief, bullying bystander intervention developed by the second author (AM) that teaches students to intervene as defenders when they witness bullying [30]. STAC stands for four STAC bullying intervention strategies: (1) “*S*tealing the show”—using humor or distraction to interrupt the bullying situation, removing the attention away from the target, (2) “*T*urning it over”—informing an adult about the bullying and asking for help, (3) “*A*ccompanying others”—befriending or providing support to the targeted student, and (4) “*C*oaching compassion”—gently confronting the perpetrator to increase empathy for the target. The STAC intervention consists of a 75-minute training during which students learn about bullying, negative consequences associated with bullying, different bystander roles, and the 4 STAC strategies that students can use to intervene when they witness bullying. Students also participate in role-plays to practice using the 4 STAC strategies. The STAC training is followed by two 15-minute booster sessions. In our prior research, we have demonstrated positive effects of the in-person STAC intervention on mental health risks and bullying victimization and perpetration in both randomized controlled trials (RCTs) and single-group, pre-post design studies. Samples in these studies included elementary, middle, and high school students enrolled in schools in affluent urban communities and low-income, rural communities, with predominantly White or Hispanic students. RCT results indicate that STAC-trained students report reductions depressive symptoms compared with control students (*β*=–.23; *P*<.01) [31]; (η^2^_P_=0.08; *P*<.02) [32]. One RCT, however, failed to demonstrate group differences on depression (η^2^_P_=0.01; *P*=.56) [33]. Findings from RCTs also indicate that students trained in the STAC intervention report greater reductions in self-reported bullying perpetration (η^2^_P_=0.08; *P*<.001) [34] and in self-reported bullying victimization (η^2^_P_=0.10; *P*<.01) [35] than control students. Findings from these studies, however, are specific to the self-reported bullying victimization or perpetration of STAC-trained students and not to students defended by STAC-trained students.

### The STAC-T App

STAC-T (Stealing the Show; Turning it Over; Accompanying Others; Coaching Compassion; Technology Version) is an online translation of the in-person STAC intervention [30]. STAC-T is a web-based modular app designed to reduce required implementation resources, provide delivery flexibility, and be easily disseminated to large groups of students who can access the intervention from a computer, tablet, or smartphone. The initial 40-minute interactive training is followed by a 15-minute booster session. The 40-minute interactive training uses knowledge checks and personalized feedback to provide education about bullying and cyberbullying, consequences of bullying, reasons students bully, bystander roles, and a description of the 4 STAC strategies. The experiential training is interactive, allowing the user to select avatars to use the STAC strategies to respond to bullying scenarios. The STAC-T training is followed by a 15-minute interactive booster session to reinforce student learning through virtual role-plays.

To assess the feasibility [36] and short-term outcomes (A Midgett and colleagues, 2026, unpublished data) of the STAC-T app, our research team conducted a large, multisite RCT with an ethnically diverse group of middle school students (N= 613) recruited from 6 low-income, rural middle schools in 3 states in the Northeast, South, and Northwest regions of the United States. We used block randomization to randomly assign classroom periods to either the STAC-T intervention or assessment-only control condition. There were a total of 29 periods, with 14 randomly assigned to the STAC-T intervention condition (n=308) and 15 to the control condition (n=305) across all 6 participating middle schools, resulting in a 1:1 allocation. Outcomes were assessed at baseline and a 30-day follow-up. Students completed the baseline data collection, STAC training, booster session, and follow-up data collection in 4 classes. Assent, study questionnaires, the STAC-T intervention, and STAC-T booster were all completed online. Of the 1802 students invited to participate in the study, 736 (40.8%) received parental consent, and of these, 613 (34.0%) also provided assent. Retention was 80.8% (249/308) in the intervention condition and 84.6% (258/305) in the control condition.

Findings demonstrated that students in the intervention condition indicated the STAC-T app had high levels of acceptability (eg, useful and interesting) and relevance [36] and reported greater increases in knowledge and confidence to intervene in bullying situations than students in the control condition at a 30-day follow-up (Cohen *d*=0.56; *P*<.001; A Midgett and colleagues, 2026, unpublished data). Bullying victimization was also assessed, with victimization measured as participant self-reported victimization using the Bullying and Cyberbullying Scale for Adolescents [37]. Results indicated that compared with students in the control condition, student in the STAC-T intervention condition reported greater increases in knowledge and confidence to intervene in bullying situations (Cohen *d*=0.56; *P*<.001; A Midgett and colleagues, 2026, unpublished data) and greater reductions in their own self-reported bullying victimization (*P*<.01) at the 30-day follow-up (A Midgett and colleagues, 2026, unpublished data). Because the victimization variable was log transformed, the effect size was reported as a percentage rather that Cohen *d*. Specifically, the students trained in the STAC-T intervention reported a 22% (95% CI 7%-35%) reduction in their own bullying victimization relative to control students. These findings provide preliminary support for the STAC-T app. However, similar to the in-person STAC program, reductions in bullying victimization are specific to STAC-trained students. Bullying victimization as an outcome for students who are defended by STAC-trained students has not yet been tested.

### Translating STAC-T Into Routine Practice

Technology-based interventions have the potential to decrease implementation barriers experienced by schools [38]. Findings from an initial STAC-T needs assessment and STAC-T usability testing conducted by our research team indicate that although key school personnel (ie, administrators, teachers, and school counselors) expressed a strong interest in STAC-T, they also identified implementation barriers, including limited time, limited financial resources, program accessibility, and potential push back from teachers, that could impact program adoption, sustainability, and feasibility [39,40]. Similarly, our qualitative findings from a series of interviews with teachers and parents suggested that for teachers, limited time, cost, and both teacher and parent buy-in were identified as the most significant implementation barriers [41,42]. For parents, the most salient implementation barriers were cost, time, program flexibility, and parent buy-in [43]. Other implementation barriers for school-based programs identified in the broader literature (eg, lack of clear antibullying policies, priorities set by key decision makers, and competing issues [22,23]) may also be relevant to the STAC-T app. These barriers were not observed in our initial STAC-T RCT due to extensive research team support for program delivery and partnerships with schools that prioritized bullying prevention and agreed to participate in research. However, they are likely to arise during efforts to scale STAC-T broadly into schools. Proactively identifying implementation strategies to address these barriers, therefore, represents an essential first step toward translating STAC-T into routine practice in schools.

### Identifying Implementation Strategies Using “Go-Zone” Methodology

Implementation strategies represent specific methods or actions that are used to enhance program implementation outcomes including acceptability, adoption, appropriateness, feasibility, fidelity, implementation cost, penetration, and sustainability by reducing implementation barriers [44]. “Go-zone” methodology [45] has been used to identify implementation strategies that are both highly important and feasible for specific evidence-based programs. The constructs of importance and feasibility are used as they are positively associated with program acceptability and program adoption [45]. “Go-zone” methodology involves asking key stakeholders to provide ratings of importance and feasibility for a list of implementation strategies for a specific setting or intervention. Strategies are categorized into four zones: (1) zone 1: high importance, high feasibility; (2) zone 2: low importance, high feasibility; (3) zone 3: low importance, low feasibility; and (4) zone: 4 high importance, low feasibility. Strategies that are rated as both highly important and highly feasible are classified as “go-zone” strategies.

The Expert Recommendations for Implementing Change (ERIC) Project [46] developed a taxonomy of 75 implementation strategies for the health care setting. Desirable implementation outcomes, however, increase when strategies are customized to be appropriate for target populations (eg, youth) and systems (eg, school) [47]. Thus, researchers have adapted the ERIC strategies specifically for school-based interventions [48]. The School Implementation Strategies, Translating ERIC Resources (SISTER) Project used an iterative process, including review and revision by a panel of implementation and school mental health experts, which resulted in 75 SISTER strategies that were more relevant to the school setting [48]. Furthermore, researchers have identified “go-zone” (ie, high importance and high feasibility) implementation strategies (eg, SISTER) for school-based interventions [49]. Specifically, school mental health professionals and administrators rated each of the 75 SISTER strategies on the dimensions of importance and feasibility for implementing school-based evidence-based practices (EBPs) targeting social, emotional, and mental health problems. Findings resulted in 33 school-specific “go-zone” implementation strategies rated as most important and feasible for the delivery of social, emotional, and mental health programming in the school setting (see Table 1 for a list of 33 strategies).

More recently, 16 SISTER strategies have been identified as “go-zone” strategies for comprehensive, school-based bullying prevention programs (D Doumas and colleagues, 2026, unpublished data). In this study, school social workers (N=254) were asked to rate the 33 SISTER strategies on the dimensions importance and feasibility and then the 33 SISTER strategies were plotted into the 4 quadrants using “go-zone” methodology. Among the 16 strategies classified as “go-zone” strategies, visual examination of the “go-zone” plot revealed that the five most important strategies were (1) developing a detailed implementation plan or blueprint, (2) conducing ongoing training, (3) making implementation easier by removing burdensome documentation tasks, (4) obtaining and using student and family feedback, and (5) involving students, family members, and other staff. The most feasible strategy identified was distribute educational materials. To date, however, no research has identified “go-zone” implementation strategies specifically for the STAC-T intervention. Furthermore, no research has investigated how to increase the feasibility of important implementation strategies that are rated as having low feasibility (ie, zone 4 strategies) for bullying prevention programs, including STAC-T.

**Table 1:** Means, standard deviations, and zone classification for the 33 SISTER^a^ implementation strategies.

Number	Strategy	Importance, mean (SD)	Feasibility, mean (SD)	Zone^b^
1	Assess for readiness and identify barriers and facilitators	4.09 (0.88)	3.43 (0.85)	1
2	Audit and provide feedback	4.18 (0.74)	3.41 (0.86)	1
3	Conduct a local need assessment	4.05 (0.85)	3.48 (0.89)	1
4	Develop a detailed implementation plan or blueprint	4.25 (0.81)	3.54 (0.84)	1
5	Develop and organize quality-monitoring systems	4.10 (0.79)	3.41 (0.84)	1
6	Develop instruments to monitor and evaluate core components of the innovation or new practice	4.01 (0.82)	3.08 (0.88)	4
7	Obtain and use student and family feedback	4.23 (0.77)	3.41 (0.92)	1
8	Monitor the progress of the implementation effort	4.05 (0.70)	3.32 (0.80)	1
9	Facilitation or problem-solving	3.88 (0.81)	3.37 (0.80)	1
10	Provide practice-specific supervision	3.59 (0.98)	2.74 (0.95)	3
11	Promote adaptability	4.09 (0.84)	3.43 (0.87)	1
12	Build partnerships (ie, coalitions) to support implementation	3.88 (1.01)	3.25 (0.90)	4
13	Capture and share local knowledge	3.54 (1.00)	3.25 (0.97)	2
14	Develop academic partnerships	3.00 (1.15)	2.72 (0.96)	3
15	Identify and prepare champions	3.43 (0.96)	2.99 (0.91)	3
16	Identify early adopters	3.51 (0.98)	3.32 (0.93)	2
17	Model and simulate change	3.72 (1.02)	3.27 (0.99)	2
18	Visit other sites	3.48 (1.05)	2.91 (1.11)	3
19	Change or alter the local environment	3.62 (0.98)	2.77 (1.02)	3
20	Conduct educational meetings	3.94 (0.91)	3.25 (0.94)	4
21	Conduct educational outreach visits	3.74 (0.93)	3.02 (1.04)	3
22	Conduct ongoing training	4.13 (0.78)	3.32 (0.98)	1
23	Create a learning collaborative	3.64 (0.96)	3.01 (0.86)	3
24	Develop educational materials	3.96 (0.92)	3.39 (1.02)	1
25	Distribute educational materials	3.92 (0.88)	3.91 (0.96)	1
26	Make training dynamic	4.08 (0.88)	3.48 (0.93)	1
27	Provide ongoing consultation	3.88 (0.91)	3.18 (0.96)	4
28	Use train-the-trainer strategies	3.79 (0.90)	3.44 (1.00)	2
29	Develop resource-sharing agreements	3.54 (0.99)	3.22 (0.96)	3
30	Facilitate relay of intervention fidelity and student data to school personnel	3.75 (0.91)	3.16 (0.93)	3
31	Make implementation easier by removing burdensome documentation tasks	4.31 (0.83)	3.48 (1.10)	1
32	Intervene or communicate with students, families, and staff to enhance uptake and fidelity	4.05 (0.95)	3.21 (0.93)	3
33	Involve students, family members, and other staff	3.96 (1.03)	3.32 (1.00)	1

^a^School Implementation Strategies, Translating ERIC Resources.

^b^Zone 1=high importance, high feasibility; zone 2=low importance, high feasibility; zone 3=low importance, high feasibility; and zone 4=high importance, low feasibility.

### This Study

The purpose of this study was to identify zone 1 (ie, “go-zone”) and zone 4 implementation strategies specific to the STAC-T app. We were also interested in how to increase the feasibility of zone 4 strategies so that schools can use these important strategies more easily to support the STAC-T intervention. To our knowledge, this is the first study to examine zone 4 strategies in this way for school-based interventions in general or specific EBPs. The study had the objectives to (1) identify “go-zone” and zone 4 strategies for the STAC-T app and (2) understand how to increase the feasibility of zone 4 strategies. To achieve these objectives, we used a mixed methods design. We used quantitative data to identify “go-zone” and zone 4 strategies and then used qualitative data to enhance our understanding of the zone 4 strategies identified by participants. First, we collected survey data from a national sample of school counselors and social workers (N=195). We used “go-zone” methodology to classify implementation strategies into 4 zones based on ratings of importance and feasibility to identify zone 1 “go-zone” strategies (ie, high importance and high feasibility) and zone 4 strategies (ie, high importance and low feasibility). We then selected a subsample (n=14) using stratified, random sampling to complete a qualitative interview to gather feedback on how to increase the feasibility of zone 4 strategies. Our primary research questions were “What are the “go-zone” strategies for the STAC-T app?,” “What are the Zone 4 strategies for the STAC-T app?,” and “How can feasibility be increased for Zone 4 strategies?”

## Methods

### Participants

We recruited a national sample of school counselor and school social workers (N=195) through the American School Counselor Association (ASCA) and the School Social Work Association of America (SSWAA). The sample was a self-selected, convenience sample. All members of ASCA and SSWAA were eligible to participate. Surveys were sent to SSWAA and ASCA members through emails and newsletter announcements. There are approximately 43,000 ASCA members and approximately 3,000 SSWAA members. Our sample was determined by budgetary constraints. All members of ASCA and SSWAA received a link to the survey and we closed it after we received 240 responses. Of these, 3 surveys were duplicate responders and we excluded the second completed survey for these participants. A total of 237 SSWAA members and ASCA members accessed the survey, consented to participation, and began the survey. Of these participants, a total of 195 (82.3%) completed the survey (Figure 1).

**Figure 1:**
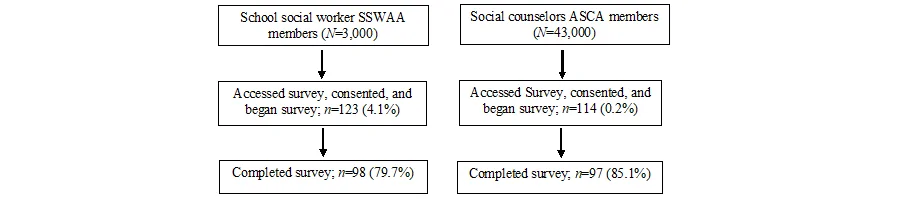
Participant flow diagram: ASCA: American School Counselor Association; SSWAA: School Social Work Association of America.

The majority of participants were female (n=177, 90.8%). Participant ages ranged from 23 to 70 (mean 40.08, SD 10.24) years. Participants self-reported ethnicity or racial background as White (n=136, 69.7%), Hispanic or Latino (n=22, 11.3%), Black or African American (n=19, 9.7%), Asian American (n=5, 2.6%), Pacific Islander (n=3, 1.5%), and other (n=10, 5.2%). Years of experience as a school social worker (n=98, 50.3%) or school counselor (n=97, 49.7%) ranged from 1 to 33 (mean 8.92, SD 7.68) years; 31.9% (n=62) reported working in more than 1 level of school, with 54.9% (n=107) working at the elementary school level, 40.5% (n=79) working at the middle school level, 12.8% (n=25) working at the junior high level, and 42.1% (n=82) working at the high school level. Size of school or district enrollment ranged from 45 to 9000 (mean 887.22, SD 991.23), with 61.5% (n=120) reporting working in an urban county and 32.8% (n=64) reporting working in a rural county. The majority of the participants (n=122, 62.6%) reported working in a title I school. Among participants, 69.7% (n=136) reported that bulling was a moderate or major problem at their school and 76.4% (n=149) reported that at least 1 student reported bullying to them in the past month. There were no differences between school social workers and school counselors on any of the demographic or bullying variables.

### Procedure

All study procedures occurred during the spring of 2024. School counselors were recruited through ASCA and school social workers were recruited through SSWAA. ASCA sent study information and a link to an online informed consent form and a survey link through the ASCA newsletter; SSWAA sent an email to all members. Participants clicked the link to log onto the study survey, which provided a welcome screen with study information and the informed consent form. Participants provided consent by clicking “Agree.” The survey took approximately 25-30 minutes to complete. At the end of the survey, participants were asked to provide their email address to receive the study incentive and to be contacted if selected for the interview. A subsample of 14 participants were selected using stratified, random sampling by profession (ie, school counselor and school social worker) and school setting (ie, rural and urban). Participants were contacted by email to participate in the interview. Participants were asked to provide information about their perceptions of the 4 zone 4 strategies that the researchers identified from the survey. Perceptions were related to (1) importance of the strategy, (2) barriers to strategy implementation, and (3) ways to reduce implementation barriers for the strategy. All individual interviews lasted 30-40 minutes and were recorded.

### Ethical Considerations

All study procedures were approved by the Boise State University Institutional Review Board (IRB; protocol number IRB23-463) and by the School Districts or Administration. We used active informed consent for this study. Participants were provided with an informed consent form that contained information about the study including purpose and background, procedures, risks or discomforts, extent of confidentiality (eg, participants were provided with a unique study identification number and study data were deidentified), benefits, payment, and how to contact the primary investigator and the Boise State University IRB. Incentives included a US $50 Amazon gift card for participation in the survey and an additional US $50 Amazon gift card for participation in the interview.

### Measures

#### Demographic Survey

Participants completed a brief demographic survey that included questions about gender, age, race or ethnicity, years of experience as a school counselor or school social worker, school level worked in, and information about their school (ie, state, county, size of school, school setting, and title I status).

#### Experiences With Bullying

Participants were asked, “How much of a problem is bullying at the school where you work?” with response choices ranging from 1 (*not a problem*) to 4 (*major problem*) and “Did students report bullying to you within the past month?” with response choices *Yes*/*No*.

#### Implementation Strategies

The School Implementation Strategies Survey [49] was used to identify zone 1 (ie, “go-zone”) and zone 4 implementation strategies [49]. Participants were given the instructions “The following questions will assess your perceptions of the importance and feasibility of specific implementation strategies related to the STAC-T app.” Implementation strategies were defined as “methods or techniques that are used to enhance the uptake, use, and sustainment of evidence-based practices (EBPs).” Participants were then given a description of the STAC-T intervention as follows:

STAC-T is a technology-based, brief bystander bullying intervention for schools, designed to teach students how to intervene in bullying situations as “defenders” using four strategies: “stealing the show” with humor or distraction, “turning it over” to an adult, “accompanying others” and “coaching compassion.” The e-training is delivered in modules, including a didactic training component, experiential activities (e.g., e-role plays with avatars), and booster activities designed to reinforce learning and maintain skills. The interactive program incorporates assessment and personalized feedback, as well as tests of knowledge, designed to increase engagement and promote learning and behavior change. The student-centered design is sensitive to the cultural and linguistic needs of students and facilitates the identification of personally appropriate strategies students can use to intervene when they witness bullying. Students can log into the app during classroom time or be directed to complete the program outside of the school setting.

Next, participants rated the importance and feasibility of each of the 33 SISTER school-based implementation strategies with regard to the STAC-T intervention. Importance was defined as “the impact of the strategy and how critical it is to a successful implementation effort.” Feasibility is defined as “the extent to which a strategy is practical and can be successfully used to support implementation.” Each implementation strategy was accompanied by a detailed definition of the strategy. For example, the strategy “Assess for readiness and identify barriers and facilitators” is defined as:

Assess various aspects of the school context to determine the degree to which it and the school personnel within it are ready to implement, barriers that may impede implementation, and strengths or facilitators (such as, coaches, professional learning communities, whole staff training) that can be used/leveraged in the implementation effort.

Response options for importance were 1 (*relatively unimportant*) to 5 (*extremely important*). Response options for feasibility were 1 (*not at all feasible*) to 5 (*extremely feasible*). The implementation strategies were developed through an iterative process of review and revision by a panel of experts in implementation and school-based mental health. Multidimensional scaling and hierarchical cluster analyses support 4 quadrants for implementation strategies in health [45], with this methodology replicated to identify 33 strategies specifically for the school setting [49].

#### Interview Questions

Participants were asked a series of open-ended questions for each of the zone 4 implementation strategies related to strategy importance, barriers to implementing the strategy, and ways to address identified implementation barriers specific to STAC-T. Participants were asked: (1) What makes this strategy important? (2) What are the barriers to implementing this strategy? (3) What could reduce those implementation barriers for this strategy?

### Data Analysis

#### Quantitative

All analyses were conducted using SPSS (version 29.0; IBM Corp). We calculated descriptive statistics for importance and feasibility ratings for each of the 33 SISTER strategies. Ratings for each strategy were examined for normality. The 33 SISTER strategies were plotted on a graph according to importance and feasibility ratings, producing a scatterplot divided into 4 quadrants or zones (eg, high importance, high feasibility; high importance, low feasibility; low importance, low feasibility; and low importance, high feasibility) using the mean of each dimension as the midpoint of each axis [45]. Zone 1 (ie, “go-zone”) represents strategies with ratings above the mean on both importance and feasibility. Zone 4 represents strategies with ratings above the mean on importance and below the mean on feasibility.

#### Qualitative

Two research team members conducted the individual interviews and transcribed the interviews verbatim. Three data analysts organized, analyzed, and described the data using thematic analysis [50-52]. A faculty member along with 2 graduate students enrolled in a Master’s in Counseling Program analyzed the data. All analysts had previous experience with qualitative methodologies and data analysis process. The faculty member led the analysis team. The lead analyst provided team members with a data analysis plan and timeline at the outset of the process and answered the team’s questions related to the data analysis process. As a first step, each team member reviewed all transcripts and analyzed the data individually, then they met in person to share their initial codes (ie, summary of participants responses) and worked to achieved consensus through discussion, resolving disagreements by referring back to participant’s quotes. Next, the analysts developed themes for each interview question by grouping codes into broader categories, then looking for differences and similarities across themes, and merging and splitting themes as needed [50]. Next, the team continued to reflect on the data analysis and continued to engage in the analysis process through several email communications across a 2-week period to achieve final consensus. The team deidentified interview data to maintain participants’ anonymity. As a next step, the data and themes were analyzed by an external auditor. The auditor agreed with the teams’ interpretation and representation of the data.

## Results

### Quantitative Data

The 4 zones for “go-zone” mapping were created using the mean of each dimension as the midpoint of each axis. Mean importance ratings ranged from 3.00 to 4.31 (mean 3.85, SD 0.29). Mean feasibility ratings ranged from 2.72 to 3.91 (mean 3.26, SD 0.25). Means, SDs, and zone classification of the 33 SISTER implementation strategies are presented in Table 1. Among the 33 SISTER implementation strategies evaluated, 15 were categorized as “go-zone” strategies (see zone 1 strategies in Figure 2). Visual examination of the “go-zone” strategies in Figure 2 indicates that the three most important and feasible strategies were (1) *making implementation easier by removing burdensome documentation tasks* (number 31), (2) *developing a detailed implementation plan or blueprint* (number 4), and (3) *obtaining and using student and family feedback* (number 7). The most feasible “go-zone” strategy *was distribute educational material* (number 25). As seen in Figure 2, 4 strategies were classified in zone 4. These strategies were (1) *develop instruments to monitor and evaluate core components of the innovation/new practice* (number 6), (2) *conduct educational meetings* (number 20), (3) *build partnerships to support implementation* (number 12), and (4) *provide ongoing consultation* (number 27).

**Figure 2:**
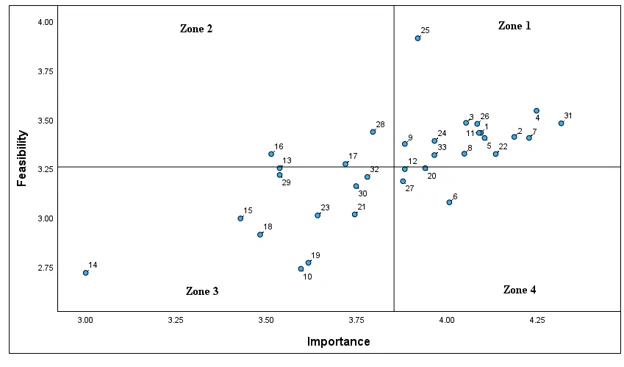
Implementation strategies for STAC-T (Stealing the Show; Turning it Over; Accompanying Others; Coaching Compassion; Technology Version) by zone. The figure depicts 4 quadrants, or zones, plotted for importance and feasibility of the 33 SISTER (School Implementation Strategies, Translating ERIC Resources) implementation strategies.

### Qualitative Data

Qualitative feedback supported the high level of importance of the zone 4 strategies identified through the survey for implementation of the STAC-T intervention and provided insights related to the relevance of the zone 4 strategies. Additionally, participants shared thoughts about barriers to implementation that reduced strategy feasibility, followed by ideas about how these implementation barriers can be addressed to increase implementation feasibility. Results are presented below, organized by each of the Zone 4 strategies.

#### Develop Instruments to Monitor and Evaluate Core Components of the Innovation or New Practice

Participants indicated that this strategy was important because it promotes fidelity and consistency in program implementation and measures effectiveness. For example, one participant shared “making sure that all the staff members are using the tool the same way, that they have that fidelity piece of it” as being of significant relevance to this strategy. Another participant shared, “You can have a fabulous program, you can develop wonderful instruments, but then if you’re not monitoring the success or ineffectiveness of it, you don’t know if it’s working or not working, if it’s reaching the kids, if it’s not reaching the kids.” Furthermore, participants talked about time, selecting a designated person to complete the task and develop a survey, and staff buy-in as potential barriers. One participant indicated, “the main barrier would just be finding the time to actually evaluate it.” Another participant contemplated, “It’s going to be difficult to find, I mean, schools don’t have enough staff as it is. So, how are we gonna add yet another piece to a parent or a teacher or administrative staff? How do we do the evaluation because who do we give that work to?” A third participant added, “Sometimes we struggle with getting buy-in for new things [STAC-T].” In terms of reducing barriers, participants talked about the importance of keeping the assessment short and simple, having the assessment be built into the STAC-T program modules with an automatically generated report for the school, and having specific school personnel assigned to the task. For example, one participant suggested, “Having...it be something really short and quick and easy to do. Like keeping it short to 2 or 3 questions” as a potential solution. Another one shared, “I think if it’s already something built-in. Like if the [STAC-T] modules were being viewed...So it’s not extra steps but it’s already included in a report [automatically generated by the STAC-T program].” A third one added, “If there was a specific team that led and did this...and if they weren't pulled into a thousand different directions...I think that could be super helpful.”

#### Conduct Educational Meetings

Participants indicated that this strategy was important to gather input and to foster a universal understanding among all stakeholders that bullying is a problem at the school. For example, a participant stated, “I think it's good to communicate what we’re doing at school to address bullying if it’s happening and to get input from different stakeholders.” Another participant shared, “I think it’s important that it’s not just the school, is not just, oh we know what’s going on, or the students, that everyone including the community, the family—that’s like the biggest—that they are always in the loop, that they’re always being educated on what’s going on in the school.” Participants discussed time and logistics as potential barriers to this strategy. A participant indicated, “some barriers are having the time and the ability to get everyone together.” A second one added, “It just can be difficult, different work schedules or lack of transportation, just tons of their own set of barriers.” However, to reduce identified barriers, participants suggested using various modes of communication, including flexibility, technology, and using a variety of tools for disseminating information. For example, a participant stated, “...a lot of different modes of delivering those educational pieces, whether it’s meetings or whether it’s communication.” Another one indicated, “You might offer a virtual option because some people can attend virtually.” And, a third one recommended, “Having different tools to share with everyone involved. So, if people are not able to be part of the meeting, sending home things that are being worked on, or a little information like that.”

#### Build Partnerships (ie, Coalitions) to Support Implementation

Participants indicated that this strategy was important in order to communicate a cohesive message to students and to have buy-in from all stakeholders. One participant indicated, “I think...involving as many, outside partners [including] after school activities or sports teams, things like that would be beneficial because if there’s bullying happening, it’s happening across all those settings. So, I think having everyone on board with the same vocabulary and the same expectations would be helpful.” Another participant shared, “...You’ve got to have a team that is going to be ready and excited to participate in it so then they can engage with the students.” In terms of barriers, participants in rural areas indicated limited accessibility to potential partnerships. For example, one participant mentioned, “We’re a very rural school. So, we don’t have a ton of partnerships...when I was thinking about who we could partner with, I came up with blanks. I couldn’t really think of, oh, we would partner with them to do this.” Participants in urban areas talked about commitment and buy-in. One participant expressed worry about, “just the overall commitment when you try and put a group together. It seems like it’s hard to get people to be consistent.” Another one talked about how “all those organizations already have their own things that they’re doing, so kind of getting that buy-in to something that another group is doing can be difficult.” In general, participants talked about schools being understaffed and school personnel being overworked. For example, a participant identified “staffing” as a key barrier, saying that “...with our school, we’re gonna have some budget cuts and staffing cuts, and people are already feeling overworked.” To reduce barriers for this strategy, participants suggested school administration setting an expectation for participation, using professional development (PD) time, and potentially providing a stipend to incentivize participation. One participant stated, “I think a strategy to reduce the barrier is to get either the principal or the supervisor of school counselors on board so it’s coming from the central office and going out. So that, it’s basically like this is what we’re doing...and it’s not a choice.” Another broached the idea of a designated PD time and space, saying, “in our school system we actually make time once a month to do what’s called a PLC, professional learning community. Just really,...if I’m able to brainstorm, to have...the time to do that without being pulled.” A third participant suggested, “The only thing...I can think of...to help facilitate implementation and somebody having to do a little bit more than maybe what’s in their job description, is extra pay.”

#### Provide Ongoing Consultation

Participants indicated that this strategy was important to support implementation and promote best practices. One participant stated, “troubleshooting or problem solving as things come up, and I think it's preferred to do it with an expert who can give you that guidance.” Another participant talked about best practices, “I know exactly what I’m supposed to be doing or what the best strategy is if something goes wrong.” However, participants indicated lack of time and identifying experts as potential barriers. One participant shared, “Figuring out a time to do that...This is so important, but I think sometimes there’s other things that might come first.” Another one stated, “Finding people who are gonna be those coaches...Finding someone who wants to take on one more thing in the school day.” To reduce barriers, participants recommended hybrid or virtual meetings, using PD time, and appointing a specific school personnel to be in charge. A participant shared that using, “Zoom meeting where everyone could be on board and attend some of the consultations.” A second one stated, “Getting to the schools before they made their schedule. Like getting put in as that PD day or getting put in that consultation day.” And a third one shared, “reaching out to people, maybe trying to get the teachers, or the people who are those support people, to be people who are outside the norm of the usual people who are being asked to do stuff.”

## Discussion

### Principal Findings

This is the first study to investigate the importance and feasibility of a set of school-based implementation strategies for the STAC-T app. We first examined the importance and feasibility of the 33 SISTER implementation strategies and then used “go-zone” methodology [45] to plot the strategies into 4 quadrants based on these 2 dimensions. Overall, we identified 15 implementation strategies in zone 1 (eg, “go-zone” strategies) and 4 strategies in zone 4 (high importance and low feasibility). Qualitative data enhanced the interpretation of quantitative findings exploring participants’ feedback about the importance of the zone 4 strategies and how to reduce barriers to increase implementation feasibility for these important strategies.

### Zone 1 (“Go-Zone”) Strategies

Using “go-zone” methodology, we identified 15 zone 1 (eg, “go-zone”) implementation strategies. The 3 most important “go-zone” strategies were (1) *make implementation easier by removing burdensome documentation tasks*, (2) *develop a detailed implementation plan or blueprint*, and (3) *obtain and use student and family feedback*. These strategies were among the most important “go-zone” strategies identified in research on comprehensive, school-wide bullying prevention programs (D Doumas and colleagues, 2026, unpublished data). Similarly, the most feasible “go-zone” strategy was *distribute educational material* both in this study and prior research on comprehensive, school-wide programs (D Doumas and colleagues, 2026, unpublished data), suggesting that the most important and feasible “go-zone” strategies may be similar for bullying prevention programs, regardless of modality of program delivery (eg, in-person and online).

Although there is considerable overlap in the “go-zone” strategies identified for STAC-T and comprehensive, school-wide bullying prevention programs, there are also important differences. For example, although *monitoring the progress of the implementation effort* was identified as a “go-zone” strategy for STAC-T, this strategy was categorized as a zone 4 strategy comprehensive, school-based bullying prevention (D Doumas and colleagues, 2026, unpublished data). The difference in zone categorization for this strategy is likely due to differences in implementation feasibility related to the complexity of monitoring implementation of an in-person program with multiple components relative to the ease of monitoring implementation of an online program. In contrast, *conducting educational meetings* was identified as a zone 4 strategy for STAC-T but a “go-zone” strategy for comprehensive, school-wide bullying prevention programs. It is possible that the differences in perceived feasibility are due to participation of school personnel in comprehensive, school-wide in-person bullying prevention, setting an expectation that school personnel would be required to attend educational meetings vs school personnel expectations related to participating in educational meetings for an online program specifically for students. These differences in perceived feasibility highlight the importance of identifying “go-zone” strategies that are specific to the STAC-T app.

### Zone 4 Strategies

Using “go-zone” methodology, four strategies were classified in zone 4 (ie, high importance and low feasibility): (1) *develop instruments to monitor and evaluate core components of the innovative or new practice*, (2) *conduct educational meetings*, (3) *build partnerships to support implementation*, and (4) *provide ongoing consultation*. With the exception of *conduct educational meetings*, these zone 4 strategies were also identified as zone 4 strategies for comprehensive, school-wide bullying prevention programs (D Doumas and colleagues, 2026, unpublished data). Next, we used qualitative data from a subsample of participants to better understand the importance of each zone 4 strategy, barriers that contribute to low feasibility, and how feasibility could be increased. Qualitative data supported quantitative data, verifying that these 4 strategies are important for successful implementation. Participant feedback also enhanced the quantitative data, suggesting that these strategies support implementation by promoting implementation fidelity, monitoring program effectiveness, creating a shared understanding that bullying is a problem, building stakeholder buy-in, and promoting best practices. Expanding our understanding of why these strategies have low feasibility, participants identified time, logistics, staff buy-in, and limited accessibility to potential partnerships and experts as implementation barriers. These barriers are consistent with those identified in our prior research [39-43] with 1 exception; participants in this study did not identify cost or limited financial resources as implementation barriers to any of the zone 4 strategies. Finally, participants provided feedback for improving the feasibility of zone 4 strategies, including keeping evaluative assessments short and simple, using various modes of communication for educational meetings, building partnerships with support from school administration, and enhancing consultation through hybrid or virtual meetings and appointing a leader. Thus, qualitative data supported the quantitative findings, enhanced our understanding of implementation barriers, and provided guidance for how to increase the feasibility of zone 4 strategies for the STAC-T app.

### Study Limitations

Although findings from this study extend the research investigating implementation strategies for the STAC-T app, there are limitations. First, although we used a national sample of school social workers and school counselors, the sample was primarily female and White, limiting the generalizability of the findings. Additionally, participants were only asked whether their school is in a rural or urban community. Thus, it is unclear whether some of the participants might have indicated suburban if that had been a response choice. Study findings are based on responses from approximately 4% of the ASCA and SSWAA members combined, further limiting generalizability. Findings should be replicated using a larger, more diverse sample, and should include additional school personnel as both administrator and teacher buy-in are important for school-based program implementation [53]. Furthermore, program implementation depends not only on school administrators and teachers but also on students, families, IT staff, and district-level administrators. Thus, these end users and key stakeholders should also be included in further implementation research.

Second, the School Implementation Strategies Survey does not specify whether respondents are to rate the 33 SISTERS items on and feasibility and importance for themselves or for other school personnel, researchers, program developers, or external partners. As no role is specified, it is likely that respondents thought about strategies as they might be most commonly applied (and by whom) in their typical work settings. Although we used the standardized instructions for the survey, as is typical in research using this measure [46,48,49], this lack of role clarity could have led to ambiguity in the interpretation of who is responsible for implementing each strategy for both the “go-zone” mapping and the responses to the interview questions. Without this role clarity, the interpretation of feasibility ratings may be less reliable than if roles were clearly defined. Further as feasibility ratings determine “go-zone” and zone 4 strategy classification, findings from this study should be cautiously interpreted in light of the role ambiguity related to strategy delivery. Role distinction may be particularly important for SISTER items such as developing instruments, conducting training, creating materials, and providing consultation. Although the literature has not currently linked implementation strategies to specific roles, this is a potentially important future direction for research.

Third, we also identified a large number of “go-zone” strategies, potentially due to a ceiling effect [54]. Specifically, the low end of the range for the mean of the items was 3.00 for importance and 2.72 for feasibility on a 1- to 5-point scale, indicating a limited range of scores. This may have occurred as we examined the previously identified “go-zone” school-based implementation strategies, which are already categorized as both important and feasible. That is, the 33 SISTER strategies already represent highly important and feasible school-based implementation strategies, limiting the range of importance and feasibility scores in this study. To address this ceiling effect, and help schools with implementation planning, we identified a subset of the most important and feasible strategies within the “go-zone.”

Finally, although participants in this study identified “go-zone” strategies for the STAC-T intervention, testing whether or not these strategies improve implementation outcomes (eg, program adoption, fidelity of delivery, and program sustainability) and program outcomes (eg, rates of bullying) was beyond the scope of this study. Future research should test the efficacy of these “go-zone” strategies on both implementation and program outcomes.

### Implications for STAC-T Implementation

Findings from this study have important implications for school mental health professionals. First, this study identified a set of implementation strategies that can be used to enhance implementation of the STAC-T app. Identifying implementation strategies extends prior literature on implementation barriers by identifying specific methods or activities that can be used to improve implementation outcomes [49]. This study identified 15 “go-zone” strategies that are both important and feasible for the STAC-T intervention. School professionals involved in program implementation can create a bundle of implementation strategies by selecting from these 15 “go-zone” strategies, choosing the strategies that may be most suitable for their school to tailor implementation [49]. Alternatively, school mental health professionals could select the strategies that were identified as most important and feasible among the “go-zone” strategies. Specifically, school mental health professionals can take a lead in designing a detailed implementation plan that can then be distributed to other school personnel involved in program delivery. School mental health professionals can work with students and their families to understand their perspectives related to the implementation plan. Feedback related to the implementation plan could be collected through surveys, focus groups, or interviews with students and parents. Furthermore, supporting school personnel by reducing barriers is important for gaining staff buy-in, which is related to adoption, implementation, and sustainability [53]. Thus, finding ways to reduce paperwork, while adhering to intervention fidelity and evaluation protocols, is important for successful implementation. Finally, educational materials, including education about rates of bullying and cyberbullying, mental health risks associated with bullying and cyberbullying, and the STAC-T intervention, can be distributed to all school personnel. Distributing educational materials is relatively simple practice requiring few resources that can be used in program implementation for youth mental health [49]. Findings from this study also provide some guidance for increasing the feasibility of zone 4 strategies. Suggestions include streamlining evaluative assessments, using various modes of communication for education meetings, which could include in-person and online meetings, engaging key school administrators to facilitate partnership building, and identifying a leader to enhance consultation.

### Conclusions

This study is the first to identify a set of important and feasible school-based implementation strategies for STAC-T, a technology-based bullying bystander intervention. We identified 15 “go-zone” strategies, with 3 of these strategies rated as most important and 1 rated as most feasible. Qualitative data enhanced quantitative findings by providing participant feedback for how to increase the feasibility of 4 strategies identified as highly important but with low implementation feasibility. Findings provide important information to enhance STAC-T implementation, which has the potential to increase both implementation and program outcomes. Clarifying who is responsible for delivering each implementation strategy, however, is an important direction for future research, as implementor role may impact strategy feasibility ratings and subsequent “go-zone” classification.

## Data Availability

The datasets generated and analyzed during this study are available from the corresponding author upon reasonable request.

## Abbreviations

ASCA: American School Counselor Association
EBP: evidence-based practice
ERIC: Expert Recommendations for Implementing Change
IRB: institutional review board
PD: professional development
RCT: randomized controlled trial
SISTER: School Implementation Strategies, Translating ERIC Resources
SSWA: School Social Work Association of America
STAC: Stealing the Show; Turning it Over; Accompanying Others; Coaching Compassion
STAC-T: Stealing the Show; Turning it Over; Accompanying Others; Coaching Compassion; Technology Version
